# Ethoxyquin is neuroprotective and partially prevents somatic and autonomic neuropathy in db/db mouse model of type 2 diabetes

**DOI:** 10.1038/s41598-021-89781-5

**Published:** 2021-05-24

**Authors:** Ying Liu, Yuan Sun, Osefame Ewaleifoh, Josh Wei, Ruifa Mi, Jing Zhu, Ahmet Hoke, Michael Polydefkis

**Affiliations:** 1 Departments of Neurology, Johns Hopkins School of Medicine, Baltimore, MD USA; 2 Present Address: Liaoning Laboratory of Cancer Genomics, Department of Cell Biology, College of Basic Medical Science, Dalian Medical University, Dalian, China; 3 Present Address: Driskill Graduate Program, Northwestern University, Chicago, IL USA; 4 Present Address: Parker University, Dallas, TX USA; 5 Present Address: Jiangsu Key Laboratory for Pharmacology and Safety Evaluation of Chinese Materia Medical, Nanjing University of Chinese Medicine, Nanjing, 210023 China

**Keywords:** Diseases of the nervous system, Myelin biology and repair, Peripheral nervous system, Autonomic nervous system, Somatic system

## Abstract

Ethoxyquin (EQ), a quinolone-based antioxidant, has demonstrated neuroprotective properties against several neurotoxic drugs in a phenotypic screening and is shown to protect axons in animal models of chemotherapy-induced peripheral neuropathy. We assessed the effects of EQ on peripheral nerve function in the db/db mouse model of type II diabetes. After a 7 week treatment period, 12-week-old db/db-vehicle, db/+ -vehicle and db/db-EQ treated animals were evaluated by nerve conduction, paw withdrawal against a hotplate, and fiber density in hindlimb footpads. We found that the EQ group had shorter paw withdrawal latency compared to vehicle db/db group. The EQ group scored higher in nerve conduction studies, compared to vehicle-treated db/db group. Morphology studies yielded similar results. To investigate the potential role of mitochondrial DNA (mtDNA) deletions in the observed effects of EQ, we measured total mtDNA deletion burden in the distal sciatic nerve. We observed an increase in total mtDNA deletion burden in vehicle-treated db/db mice compared to db/+ mice that was partially prevented in db/db-EQ treated animals. These results suggest that EQ treatment may exert a neuroprotective effect in diabetic neuropathy. The prevention of diabetes-induced mtDNA deletions may be a potential mechanism of the neuroprotective effects of EQ in diabetic neuropathy.

## Introduction

Diabetic peripheral neuropathy (DPN) is a major complication of diabetes that affects predominantly sensory and autonomic axons. More than half of patients with diabetes have evidence of peripheral neuropathy that can result in neuropathic pain, sensory loss and autonomic dysfunction in the form cardiovascular problems, abnormal sweating, sexual dysfunction and gastrointestinal dysmotility^1^. The combination of sensory and autonomic abnormalities contributes to the morbidity and mortality of patients with diabetes resulting in limb amputation^2–5^, painless heart attack and sudden death^6,7^.

Although the pathogenesis of DPN is incompletely understood, several mechanisms have been implicated including oxidative stress^8^ and mitochondrial dysfunction^9^ which can be caused by mitochondrial DNA (mtDNA) mutations resulting from inefficient proofreading function of DNA polymerase gamma (Pol γ)^10,11^. Mitochondrial dysfunction can be caused by a number of mechanisms including mtDNA deletions. Previous studies have demonstrated that elevated intracellular levels of reactive oxygen species (ROS)^12–15^ can result in accumulation of mtDNA mutations that triggers a cycle of increased ROS production and further mtDNA mutations^16^. MtDNA deletions and mitochondrial dysfunction have been linked to aging and cancer^17^. Similarly, abnormalities in mitochondrial fusion and fission has been described in animal models of DPN^18^ and accumulation of mtDNA deletion mutations has been shown to play a role in HIV-associated peripheral neuropathy^19^. However, we still do not have a clear understanding of whether mtDNA mutations and subsequent mitochondrial dysfunction is involved in diabetic neuropathy.

We previously identified ethoxyquin (EQ) as a potent neuroprotective compound through a phenotypic screen against three neurotoxic drugs and demonstrated that it prevents development of peripheral neuropathy in paclitaxel and cisplatin models of chemotherapy-induced peripheral neuropathy (CIPN). EQ`s mechanism of action was linked to its ability to inhibit the chaperon domain of Hsp90 thereby reducing the cellular levels of one of its client proteins, SF3B2^20,21^. Although SF3B2 is involved in RNA splicing, how it links with molecular machinery that mediates distal axon degeneration in peripheral neuropathy is incompletely understood. In this study, we assessed the effect of EQ in a genetic model of type II diabetes, the db/db mouse^22,23^ and assessed the role that mtDNA deletion mutation might play. We previously demonstrated that db/db animals develop neuropathy by six weeks of age^24^. Thus, a study protocol with treatment starting at 6 weeks allows us to ask whether EQ can prevent progression of DPN. Here, we show that EQ demonstrates neuroprotective effects on several measures of both sensory and autonomic diabetic neuropathy and that this neuroprotection correlates with reduction in mtDNA deletion mutations induced by diabetes.

## Results

### EQ does not affect body weight and blood glucose in db/db mice

**Figure 1:**
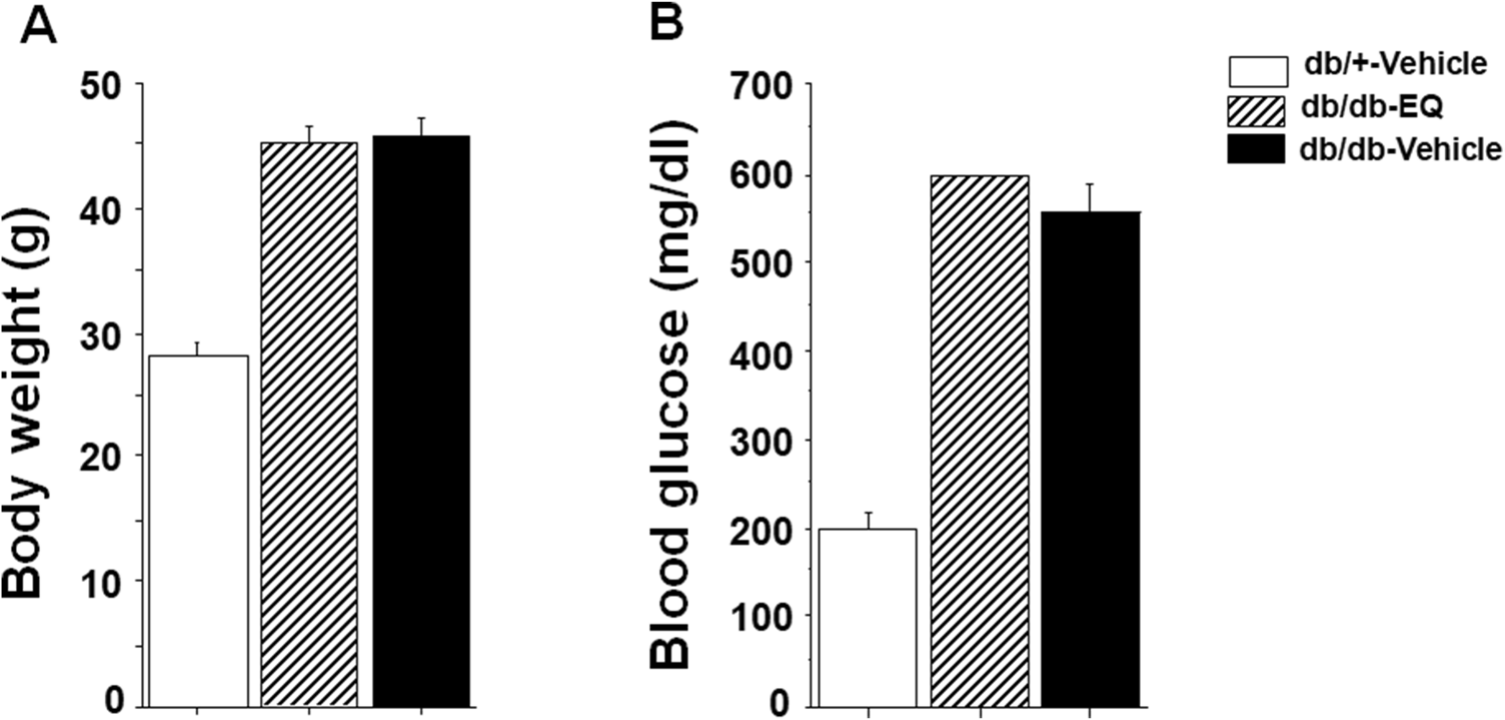
EQ does not have an effect on body weight and blood glucose. Body weight **(A)** and blood glucose **(B)** in db/+ vehicle, db/db-vehicle and db/db-EQ mice (n = 8 per group). EQ had no effect on body weight and blood glucose after seven weeks of treatment.

Weight and blood glucose levels were assessed throughout the study. After 7 weeks, weight was 45.16 ± 1.45 g in EQ treated db/db mice and 45.9 ± 1.48 g in vehicle treated db/db mice. Glucose levels were 597.7 ± 2.29 and 556.6 ± 30.1 mg/dl in EQ-treated and vehicle-treated db/db mice demonstrating that EQ administration had no metabolic effect (Fig. 1).

### Innervation of sensory and autonomic nerves fibers in diabetic mice was improved with EQ treatment

**Figure 2:**
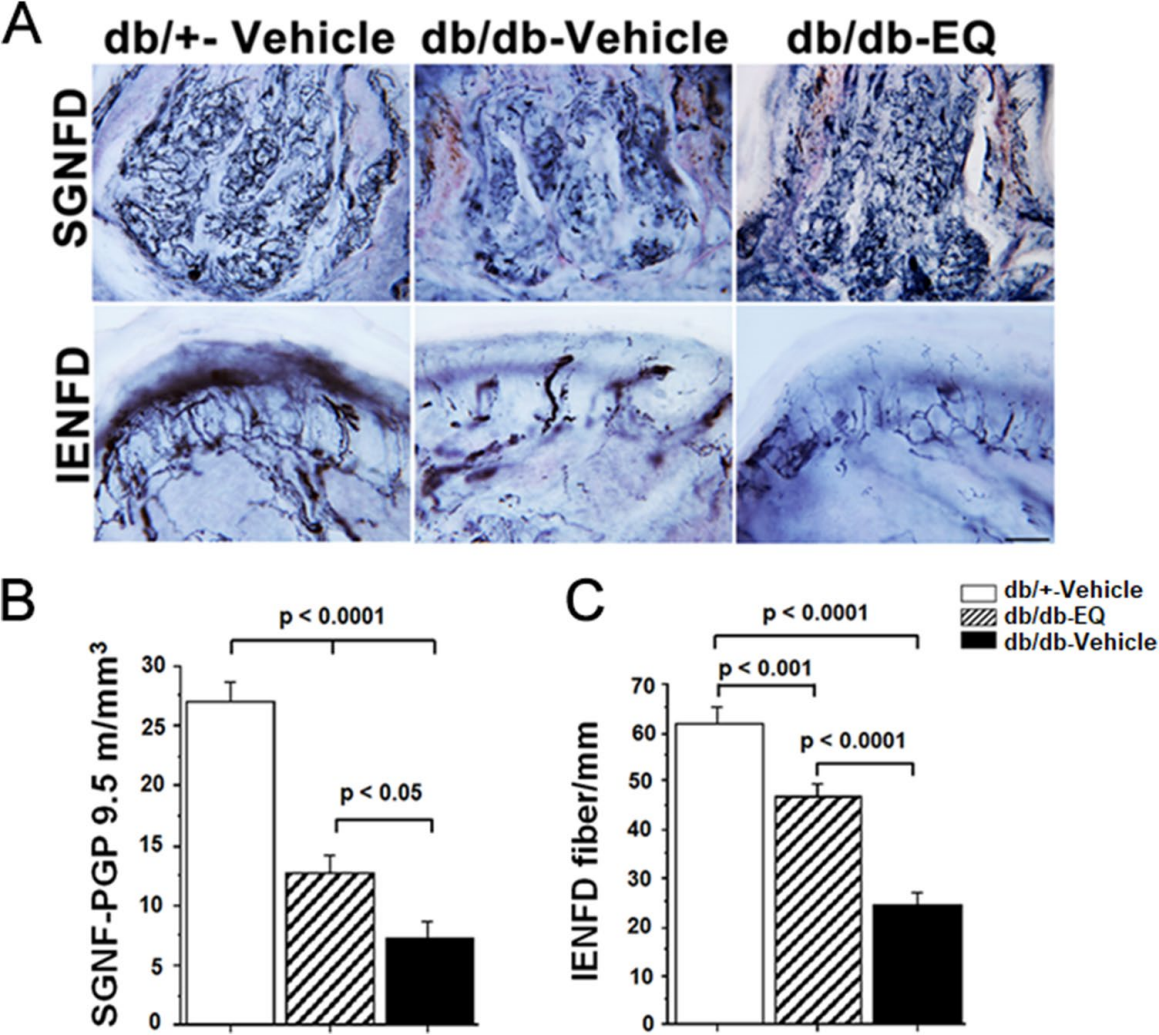
EQ treatment has a neuroprotective effect on unmyelinated sensory and autonomic fibers. **(A)** Representative images showing the innervation of sweat gland (top) and epidermis (bottom) from the footpads of db/+ -vehicle (left), db/db-vehicle (middle) and db/db-EQ (right) mice at 12 weeks of age. Nerve fibers were visualized by staining for pan-neuronal marker PGP 9.5. Innervation of footpads of db/db-vehicle mice is reduced compared with db/+ -vehicle and db/db-EQ footpads. Innervation of footpads of db/db-EQ mice is higher compared with db/db-vehicle footpads. Scale bar = 100 µm. (**B)** Quantification of PGP 9.5-postive fibers of sweat gland nerve fiber density (SGNFD) in db/+ -vehicle, db/db-EQ and db/db-vehicle mice (n = 8 per age group). SGNFD is significantly higher in db/+ -vehicle group compared to db/db-vehicle and db/db-EQ group; However, after 7 weeks EQ treatment, SGNFD in db/db-EQ mice is significant higher compared to db/db-vehicle mice (12.64 ± 1.62 m/mm^3^ in db/db-EQ group, 7.36 ± 1.46 m/mm^3^ in db/db-vehicle group, 27.1 ± 1.7 m/mm^3^ in db/+ -vehicle group). Bars represent means ± SEM. (**C)** Quantification of PGP 9.5-postive fibers of intraepidermal nerve fiber density (IENFD) in db/+ -vehicle, db/db-EQ and db/db-vehicle mice (n = 8 per age group). IENFD is significantly higher in db/+ -vehicle group compared to db/db-vehicle and db/db-EQ group; IENFD in db/db-EQ mice is also significant higher compared to db/db-vehicle mice (46.74 ± 3.0 fiber/mm in db/db-EQ group, 24.85 ± 2.3 fiber/mm in db/db-vehicle group, 61.96 ± 3.1 fiber/mm in db/+ -vehicle group). Bars represent means ± SEM.

We previously have shown that small fiber sensory and autonomic neuropathy occurs as early as six weeks of age in db/db mice^24^. The db/db mouse is an accepted genetic model of type 2 diabetes due to a mutation in the leptin receptor. The model replicates many features of type 2 diabetes including weight gain, insulin resistance, hyperglycemia, lipd abnormalities and peripheral neuropathy. Thus, in order to assess whether EQ has a neuroprotective property, treatment began at 6 weeks of age. Following 7 weeks of treatment, small unmyelinated fibers density was assessed in sweat glands (SGNFD) and epidermis (IENFD). db/+-vehicle control group IENFD was 61.96 ± 3.1 fibers/mm while vehicle-treated db/db mice had reduced IENFD at 24.85 ± 2.3 fiber/mm. This distal axon loss was partially prevented in EQ-treated db/db mice, which had an IENFD of 46.74 ± 3.0 fiber/mm. A similar pattern of neuroprotection was observed in SGNFD. In db/+ vehicle treated control mice, SGNFD was 27.1 ± 1.7 m/mm^3^ but it was reduced to 7.36 ± 1.46 m/mm^3^ in db/db-vehicle treated mice. In EQ treated db/db-EQ mice, the SGNFD was partially rescued to 12.64 ± 1.6 m/mm^3^. These findings suggest that in db/db mice, there is both somatic and autonomic denervation of distal targets and that this is partially ameliorated with EQ treatment (Fig 2).

### Treatment with EQ partially abrogates thermal heat hypoalgesia and electrophysiological dysfunction

**Figure 3:**
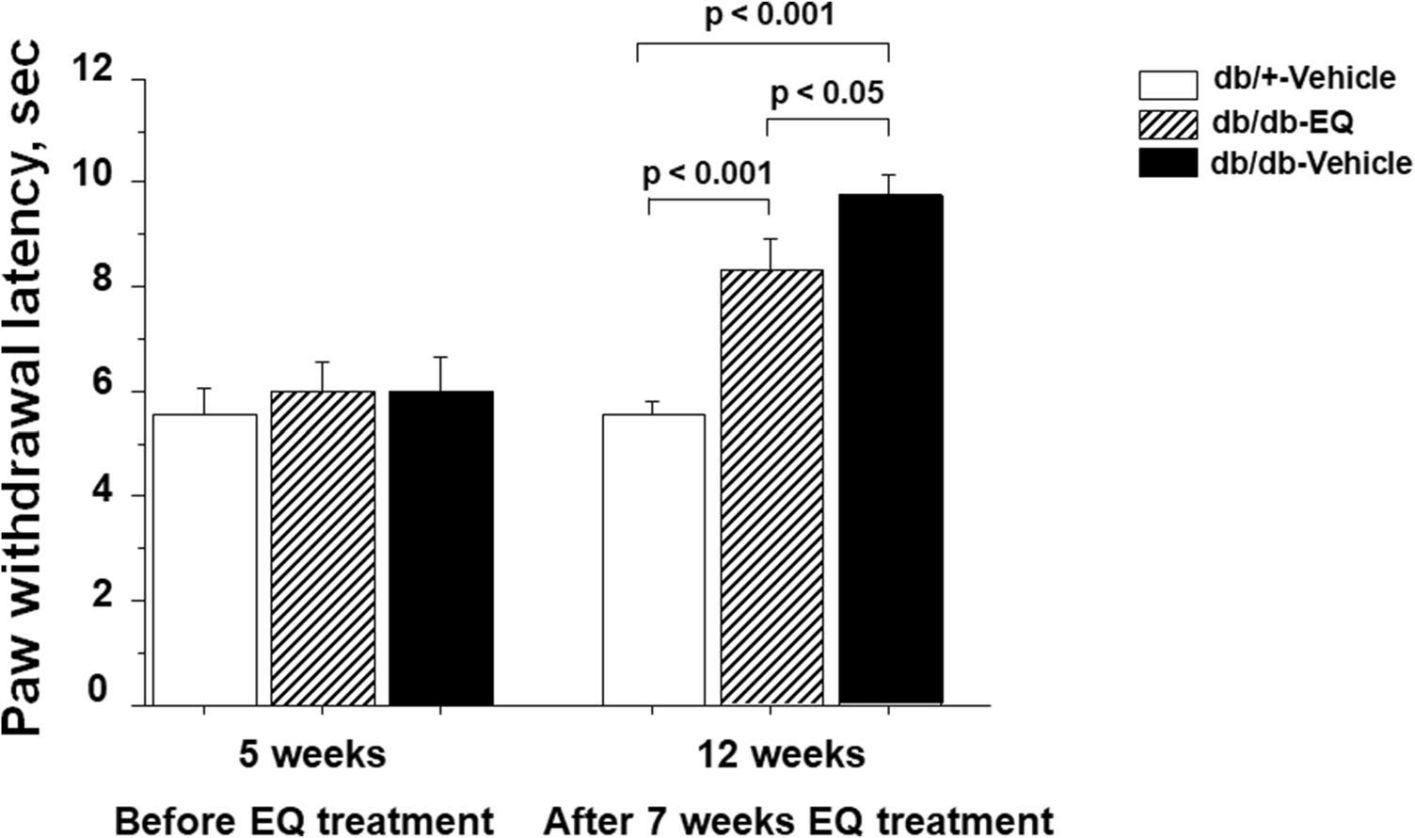
Effect of EQ treatment on paw withdrawal to a thermal stimulus. At intensity of 16%, the thermal latency was significantly delayed in db/db-vehicle mice at 12 weeks (9.74 ± 0.41 s) compared to age-matched db/db-EQ (8.29 ± 0.6 s) and db/+-vehicle mice (5.59 ± 0.28 s; n = 15 per group). The thermal latency was significantly rescued in db/db-EQ mice compared to age-matched db/db-vehicle mice. Bars represent means ± SEM.

In order to examine if there is a functional correlate of the pathological changes, we examined the paw withdrawal latency to heat stimuli at 5 weeks of age, before start of EQ treatment and at 12 weeks upon completion of the EQ treatment. At age 5 weeks of baseline, there was no latency difference between the three groups (db/+ -vehicle: 5.6 ± 0.73 s, db/db-vehicle: 6.03 ± 0.66 s and db/db-EQ; 6.02 ± 0.59 s). At 12 weeks of age, the db/+ -vehicle control mice did not show any significant change compared to 5 weeks of age (5.59 ± 0.28 s). Consistent with previous findings, the thermal latency was prolonged in the db/db- vehicle treated mice (9.74 ± 0.41 s), and this change was partially prevented by EQ treatment in the db/db- EQ mice (8.29 ± 0.6 s) (Fig. 3).

**Figure 4:**
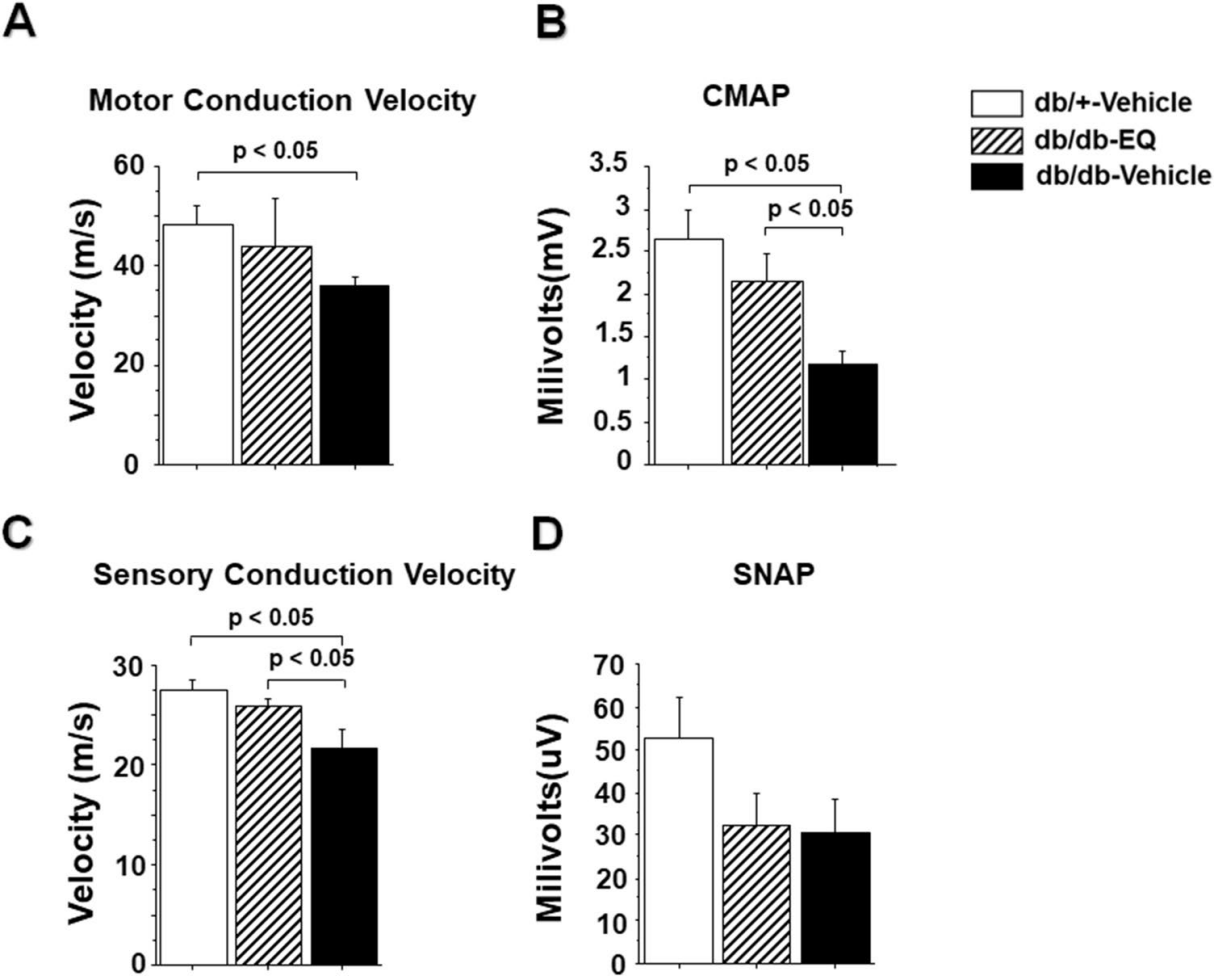
Effect of EQ on large diameter myelinated sensory and motor nerve function. (**A)** Motor nerve conduction velocity in db/db-vehicle mice was significantly decreased (35.93 ± 1.9 m/s) compared to age-matched db/+ -vehicle mice (48.22 ± 3.8 m/s), but the velocity was improved with EQ treatment (43.91 ± 9.58 m/s in db/dv-EQ mice). Bars represent means ± SEM. (**B)** Amplitudes of compound muscle action potential (CMAP) was significantly reduced in 12-week-old db/db-vehicle mice. CAMP was significantly improved in db/db-EQ mice compared to db/db-vehicle mice (2.64 ± 0.35 mV in db/+ -vehicle mice, 2.16 ± 0.33 mV in db/db-EQ mice and 1.185 ± 0.14 mV in db/db-vehicle mice. n = 6 per group). Bars represent means ± SEM. (**C)** Sensory nerve conduction velocity was significantly decreased in db/db-vehicle mice (27.58 ± 0.95 m/s) compared to age-matched db/+ -vehicle mice (21.18 ± 1.79 m/s) and db/db-EQ mice (25.84 ± 0.86 m/s). Bars represent means ± SEM. (**D)** Amplitudes of sensory nerve action potential (SNAP) in tail nerves were not statistically different between groups.

Results from the electrophysiological studies followed a similar pattern. As shown in Fig. 4, there was reduction in both motor and sensory NCV among db/db− mice compared to db/+ mice that was partially prevented in EQ treated db/db-mice (43.91 ± 9.58 m/s vs 35.93 ± 1.9 m/s in the motor nerves and 25.84 ± 0.86 m/s vs 27.58 ± 0.95 m/s in sensory nerves). This prevention of conduction velocity slowing was paralleled in the sensory and motor amplitudes. In db/db− vehicle treated mice, there was a reduction in CMAP (1.185 ± 0.14 mV) compared to db/+ control group (2.64 ± 0.35 mV) and this was partially rescued in the db/db-EQ mice (2.16 ± 0.33 mV). Although there was a reduction in amplitudes of evoked sensory nerve responses in the diabetic groups, these differences were not statistically significant.

### Increased mitochondrial DNA (mtDNA) deletion burden in db/db sciatic nerve was prevented with EQ treatment

**Figure 5:**
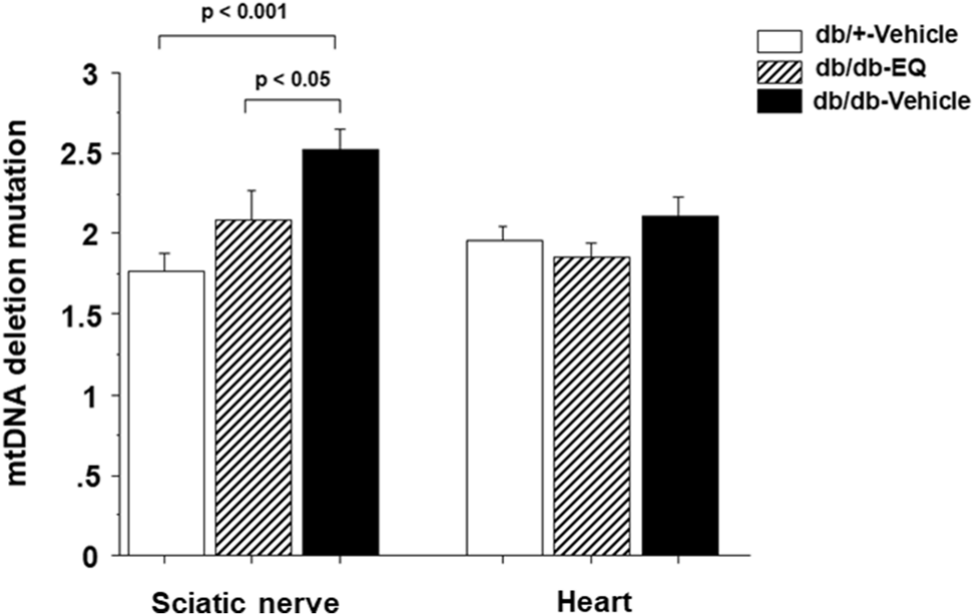
Increase in mitochondrial DNA mutation deletions in the distal end of sciatic nerve was prevented by EQ treatment. Mitochondrial DNA mutation deletion was measured in the distal end of sciatic nerve and the apical cardiac tissue in db/+-vehicle (n = 8 per group), db/db-EQ (n = 5 per group) and db/db–vehicle mice (n = 8 per group). The deletion burden in distal end of sciatic nerve was higher in db/db-vehicle mice compared with db/+ -vehicle mice at 12 weeks age. This impairment was partially prevented with EQ treatment. However, the deletion burden in the apical cardiac tissue was not different among the groups. Bars represent means ± SEM.

Mitochondrial DNA deletions have been linked to many neurological diseases including diabetic neuropathy^25^. The high metabolic demands of maintaining an electron gradient across membranes make nerves vulnerable to mitochondrial dysfunction. Peripheral nerves with their long axonal projections are particularly susceptible to energy failure. We assessed mitochondrial DNA deletions in the distal portion of sciatic nerve. The total mtDNA deletion measurement in 12-week-old db/db-vehicle mice was increased (2.57 ± 0.095 a.u.) compared to db/db-EQ (2.086 ± 0.19 a.u., p < 0.05) and db/+ vehicle mice (1.78 ± 0.084 a.u., p = NS) (Fig. 5). We also examined the mtDNA deletion burden in apical cardiac tissue between groups to see if this effect was unique to the long axons. However, unlike sciatic nerve, there was no increase in mtDNA deletion in diabetic cardiac tissue.

## Discussion

Apart from optimizing glucose control and increasing aerobic exercise^26–29^, there are no treatments to prevent progressive distal axonal degeneration seen in DPN. In this study, using a genetic model of type II diabetes, we show that ethoxyquin, a recently identified neuroprotective compound^20,21^, can partially prevent distal axonal degeneration and functional consequences of diabetes in sensory and motor nerves.

In this study, we began EQ treatment at 6 weeks because we did not see any defect in thermal sensation at 5 weeks and our prior study had shown that db/db mice develop signs of peripheral neuropathy by age 6 weeks and the defect of thermal sensation is consistent with the denervation^24^. As a neuroprotective study design, our findings show that as the disease evolves, there is evidence of an accumulated damage to both somatic and autonomic nerve fibers that can be partially prevented by EQ treatment. Although we did observe an impact on thermal sensation and electrophysiological parameters, our primary outcome measure was a direct histological evaluation of the epidermal and sweat gland innervation, where we saw about 35% rescue in IENFD and 19.5% in SGNFD. The difference in EQ`s effect on somatic and autonomic nerves might be due to autonomic neuropathy being more severe than somatic neuropathy in diabetic animal model and patients^24,38^. We observed that SGNFD and IENFD were reduced by 72.8% and 59.8% respectively in db/db mice compared to control db/+ mice at 12-weeks. Our prior study^24^ observed similar patterns with a SGNFD and IENFD reduction of 50.7% and 33% respectively compared to control db/+ mice at 6-weeks. The sweat function and thermal sensation tests are consistent with the denervation: the number of sweat droplet was reduced by 49.8% and the thermal latency was prolonged by 26% in db/db mice compared with control db/+ mice.

A limitation of this study is that we tested the efficacy of EQ at a single dose. Although 750 µg/kg/day was sufficient to provide significant neuroprotection against paclitaxel^21^ and cisplatin^20^, it is possible that with a higher dose EQ, we could achieve even greater neuroprotection in diabetes. Alternatively, the neuroprotective pathway activated by EQ may only be partially relevant for the mechanism of axonal degeneration seen in type II diabetes where the damaging effects of hyperglycemia are compounded by the effects of obesity and metabolic syndrome. Given that previous studies highlighted that obesity is a risk factor for peripheral neuropathy^30,31^, we also examined the impact of EQ treatment on body weight and glucose control in the db/db mice. Since, EQ treatment did not alter body weight and glucose levels, we can conclude that the neuroprotective effect of EQ is not an indirect one. In fact, lack of EQ’s impact on blood glucose was also shown in fish^32^. EQ is widely used as a preservative in animal feed and has been shown to be readily absorbed from the gut, and rapidly metabolized by the liver and not in sufficient quantities in blood to have any anti-oxidant effects. The oral bioavailability is estimated at 10% suggesting that oral dosing is possible in future experiements.

Although, ethoxyquin is an antioxidant, and ROS have been shown to play a role in development of DPN, it is not clear that the neuroprotection we observed is due to it`s antioxidant properties. The antioxidant properties of EQ have been demonstrated using higher concentrations in the 50–1000 µM range in vitro^33^ while the neuroprotection against paclitaxel is observed optimally around 300 nM in vitro^21^. Given that several previous studies have shown that mtDNA mutations are associated with diabetic complications^34,35^, we asked if there was an increase in mtDNA mutation burden in distal sciatic nerves of db/db mice. Using a newly developed assay that examines total mtDNA deletion burden, we found that there was an increase in mtDNA deletions in sciatic nerves but not in cardiac muscle in db/db mice. This was not seen in the EQ-treated db/db mice suggesting that broad neuroprotection afforded by EQ may act through multiple mechanisms, including correcting mtDNA deletion mutations induced by the diabetes and metabolic syndrome in db/db mice. Obviously, we do not know how EQ might be preventing mtDNA mutations caused by diabetes. The immediate neuroprotective mechanism of action of EQ in chemotherapy induced neurotoxicity appears to be related to its ability to inhibit chaperone domain of heat shock protein 90 (Hsp90)^21^. This chaperone domain inhibition is specific and results in improper folding and degradation of a client protein, SF3B2. SF3B2 is a component of the spliceosome complex and is important in mRNA splicing^36^. Therefore, it is likely that EQ-treated cells have altered RNA splicing, which may affect mtDNA replication and/or quality control. Future studies will help identify the exact mechanism of neuroprotection afforded by EQ.

In conclusion, this study confirms that peripheral nerve is affected by 6 weeks in db/db mice. The progressive functional and structural changes seen in the db/db mouse model of diabetes were partially prevented by 7 weeks of treatment with EQ. This neuroprotective effect was associated with a reduction in accumulation of mtDNA deletions and independent of any effect on glucose levels. Combined with positive results of EQ in models of chemotherapy neuropathy, these findings suggest that EQ has a neuroprotective effect that is associated with reductions in hsp90 and mtDNA deletions. Additional studies are needed to determine if higher doses of EQ or longer treatment with EQ provide better neuroprotection. Given safety profile of EQ, these findings have potential for human translation.

## Material and methods

### Animal subjects

15 db/+ and 35 db/db male mice (BKS.Cg-DOCK7 background, Jackson Labs, Bar Harbor, Maine, USA) were studies and an approved by the Johns Hopkins University Committee on the Use and Care of Animals protocol. All methods were performed in accordance with the relevant guidelines and regulations and in compliance with the ARRIVE guidelines.

Mice were treated with Ethoxyquin (EQ) or vehicle i.p. 6 days/week for seven weeks beginning at age six weeks. db/db mice were administered EQ at 750 µg/kg/day. This dose was selected based on prior experience in CIPN models. The control db/db and db/+ mice were administered with an equal volume of vehicle (5% ethanol/5% tween-80 in phosphate buffered saline (PBS)). EQ stock solution was created by dissolving 18.75 mg of EQ in 5 ml of 100% ethanol, then divided into 200 μl/vial and stored at −20 °C. The working solution was prepared just before use by mixing 200 μl stock solution, 200 μl tween-80 and 3.6 ml PBS. All mice were euthanized with CO_2_ at age twelve weeks following 7 weeks of treatment.

### Electrophysiological studies

All electrophysiology studies were performed 24 h following the last dose of EQ administration. Mice were anesthetized with isoflurane and maintained at 32 °C with a heating lamp for all studies. Motor and sensory nerve studies were performed according to standard protocols with the aid of Power Lab signal acquisition setup (AD Instruments, Colorado Springs, CO, USA)^24^. Briefly, compound muscle action potentials (CMAP) were acquired by supramaximal stimulation of the sciatic nerve while recording from intrinsic foot muscle on the plantar surface. Sciatic nerve conduction velocity (NCV) was measured by placing stimulating electrodes at proximal and distal sites: the first set of electrodes were placed at the sciatic notch and the second set of stimulating electrodes were placed at 1.7–2.0 cm distally along sciatic nerve. The distance between two sites divided by the latency difference was used to calculate sciatic NCV. Sensory nerve evoked action potentials were recorded in the tail nerve. The recording electrode was placed at the base of the tail, and the stimulating electrode was placed 5 cm distally. Sensory nerve action potential (SNAP) amplitude was recorded as the average of 20 stimulations, latency was determined, and conduction velocity was calculated. Mice that have undergone electrophysiological study were not used to assess mtDNA deletion burden in sciatic nerve to avoid electrical stimulation as an interfering factor.

### Thermal latency behavioral test

Timed latency to hind limb withdrawal was assessed at 32 °C (IITC Life Sciences Model 400 heated base, Woodlands Hills, CA). Following brief radiant heat exposure aimed at the ventral aspect of the paw from a heated lamp applied through a temperature modulated Plexiglas, thermal latency was recorded. For each mouse, 6 trials, at 20-min intervals, were scored to obtain an average response^24^. Results of all recorded latencies were averaged for each mouse and counted as n = 1 to generate a mean and SEM. Behavioral testing was performed at baseline and after 7 weeks of EQ treatment, 24 h after the last dose.

### Immunocytochemistry

Visualization of epidermal and sweat gland nerve fibers was performed according to a standardized protocol^24,37^. Briefly, mice were euthanized with carbon dioxide and hind limbs were removed and fixed in Zamboni fixative (Newcomers Supply, Middleton, WI) for 72 h, washed with phosphate buffer and then transferred in cryoprotectant (30% glycerol) solution. Tissue blocks were cut by freezing microtome at 50 µm intervals. Four sections were selected from footpads 3 and 4 and immunohistochemically stained with rabbit anti-PGP 9.5 (AbD Serotec, a Bio-Rad Company, Kidlington, UK) through a standard chromogen technique.

### Intraepidermal nerve fiber density (IENFD) and sweat gland nerve fiber density (SGNFD) analysis

PGP 9.5-IENFD and SGNFD were calculated as described^24,37^. All data collection was performed blinded to study group assignment. Briefly, IENFD was measured by the number of individual PGP 9.5 positive fibers located in the epidermis across the junction of the epidermis and the dermis. IENFD was calculated by dividing the number of counted fibers by the length of epidermis and expressed as fibers/mm. The density of PGP 9.5 positive axons in sweat glands (SGNFD) of footpads were measured by stereological length estimation using spherical probes option (based on an unbiased fractionator sampling methodology), Stereo Investigator (MicroBrightField, Williston, VT)^24,38^. Using a high-power objective lens with a high numerical aperture (× 100/1.25), the top and bottom of the footpad sections were identified, and the thickness of the mounted sections was determined. A virtual hemisphere with a dissection height of 15 µm and a guard zone of 1 µm on both surfaces was used. The largest portion of the gland contour was traced using × 10/0.30 Plan Neofluar objective of a Zeiss light microscope (Carl Zeiss Meditec, Dublin, CA). A program-generated grid ensured systematic random sampling of sites. Under × 100/1.25 at each site, a virtual hemisphere of constant volume was delivered through the z-axis plane with the stage controlled by the program. The structure was counted as a “profile” when a fiber transected the hemisphere boundary. Sweat gland volume was calculated using the Cavalieri method that we previously used for human skin biopsies^38^. All measurements were obtained using DAT files, and sweat gland innervation was expressed as m/mm^3^ for each sweat gland fragment.

### Total mtDNA deletion assay

Distal portion of sciatic nerve were utilized to examine total mtDNA deletions using a nested PCR protocol and end-labeling of amplified mtDNA fragments in 3 regions of mtDNA. To each section, 20 μl of NaOH (50 mM) was added and the tubes were kept at 95 °C for 25 min. The reaction was neutralized by adding 2.2 μl TrisHCl (pH 8.0). For PCR amplification, forward and reverse primers (Table 1) were added to a final concentration of 0.5 μM and dH2O was added to bring the total volume to 25 μl. One puReTaq Ready-To-Go PCR bead (GE Healthcare, NJ, USA) was added to each reaction and the amplifications were performed using a MJ DNA Engine Opticon 2 (Bio-Rad, California, USA). The genomic DNA were first denatured at 95 °C for 2 min, and then amplified by PCR for 20 cycles (95 °C for 30 s, 57 °C for 45 s, 72 °C for 45 s), followed by extension at 72 °C for 10 min. 2 μl PCR product from the first amplification were transferred to a new well in a 96-well plate, and amplified with nested primers (at the same concentration as in the first amplification) for 30 cycles (95 °C for 30 s, 58 °C for 45 s, 72 °C for 45 s), followed by extension at 72 °C for 10 min. For internal control, 2 μl PCR product from first PCR reaction were also amplified using a pair of primers which result in a short (300 bp) PCR product. The PCR product were then cleaned of the free primers using PCR Purification Kit (Qiagen, CA, USA). After 3 washes, PCR product were eluted in 200 μl 90 °C dH2O. 100ul of the PCR product was used for fluorescence reading in a FlexStation 3 fluorescence reader (Molecular Devices, CA, USA). Since the short DNA can be amplified much more effectively under the designed amplification conditions, the fluorescence reading represents mostly the amplification of mitochondrial DNA with large deletion(s). Ratio of this amplified PCR product fluorescence reading to internal control PCR product fluorescence reading were used to compare the quantity of the original mitochondrial genomic DNA templates with deletions. Primers used in the study were designed according to the published mouse mitochondrial genome (Table 1).

**Table 1:** Primers.

Lable name	Position	Sequence	Left or right
M1A	Mice Mt 131	GAC CGG TGT AAA ATC CCT TAA ACA	Left
M1A	Mice Mt 5174	TTT CGG CGG TAG AAG TA	Right
M1B	Mice Mt 2793	ATC GCC ATA GCC TTC CTA ACA	Left
M1B	Mice Mt 7963	TTG GGG TAA TGA ATG AGG CAA ATA G	Right
M1C	Mice Mt 7539	CAG GCC GAC TAA ATC AAG CAA CAG	Left
M1C	Mice Mt 12,963	CTC AGG CGT TGG TGT TGC AG	Right
M2A	Mice Mt 5102	TCC TGC CAA TCT AGT TGA GGT CTT A	Right
M2A	Mice Mt 169	5’-56-FAM/AAG GAG AGG GTA TCA AGC ACA TTAA	Left
M2B	Mice Mt 2855	5’/56FAM/AGGCCCT AACATTGTTGGTCCATAC-3’	Left
M2B	Mice Mt 7916	AAG GGG TTT TTA CTT TTA TGG TTGT	Right
M2C	Mice Mt 7590	5’-56-FAM/ATG GCC AAT GCT CTG AAA TTT GTGG	Left
M-CONTROL	Mice Mt 365	5’- ACA CGT TTT ACG CCG AAG AT	Right
M-5kbctrl	Mice Mt 4671–4686	GCCTTCCACCACTAACGAGA	Left
M-5kbctrl	Mice Mt 4928–4909	GGGGTAGGGTTATTGTGCTT	Right
M-5 kb	Mice Mt 8382–8401	ACCAATGGCATTAGCAGTCC	Left
M-5 kb	Mice Mt 13,489–13,469	AAGCCTTTTTGGTTGGTTGTT	Right

### Statistical analysis

The comparison between groups were made by two-way ANOVA. Pairwise comparisons were made using Student’s t-test. Prism 6.0 (GraphPad, La Jolla, CA) was used to perform the analyses and any value of p < 0.05 was scored as statistically significant. Data are presented as mean ± SEM.
